# Dietary herbaceous mixture supplementation reduced hepatic lipid deposition and improved hepatic health status in post-peak laying hens

**DOI:** 10.1016/j.psj.2022.101870

**Published:** 2022-03-24

**Authors:** Yao Zhu, Xiangli Zhang, Pengfei Du, Ziyang Wang, Pengna Luo, Yanqun Huang, Zhenhua Liu, Huaiyong Zhang, Wen Chen

**Affiliations:** ⁎ College of Animal Science and Technology, Key Laboratory of Animal Biochemistry and Nutrition, Ministry of Agriculture, Henan Agricultural University, Zhengzhou 450002, China; † Henan Jinqianguo Bio Tech Co., Ltd, Zhengzhou 477150, China

**Keywords:** herbaceous mixture, lipid deposition, liver injury, post-peak laying hen

## Abstract

Fatty liver hemorrhagic syndrome is characterized by hepatic damage and hemorrhage impairing animal welfare in birds, which was well-known to be moderately relieved through dietary choline chloride supplementation in laying hens. Chinese herb has been proven to exert a positive role on hepatic health in human and rodents. Here, we investigated the effect of herbaceous mixture (**HM**), which consists of *Andrographis paniculate, Silybum marianum, Azadirachta Indica*, and *Ocimum basilicum* (2:3.5:1:2), on the hepatic lipid metabolism and health status in laying hens. A total of 240 Hy-line Brown hens (389-day-old) were randomly fed the basal diet with 0 mg/kg choline chloride (negative control, **NC**), 1,000 mg/kg choline chloride (control, **Ctrl**), or 300 mg/kg HM for 28 d. Birds fed HM diet exhibited lower serum triglyceride (**TG**) and low-density lipoprotein cholesterol concentration, and higher high-density lipoprotein cholesterol level than those received NC and Ctrl diets (*P* < 0.05). When compared to control and NC group, the diets with HM decreased the contents of total cholesterol and TG in liver, as well as upregulated the mRNA abundance of hepatic hormone-sensitive lipase and lipoprotein lipase. Meanwhile, the hepatic area and diameter of steatosis vacuoles were also decreased by dietary HM administration (*P* < 0.05), which accompanied by decreased serum alanine aminotransferase activity (*P* < 0.05). Birds fed HM diets enhanced the hepatic antioxidative capacity than those received NC and Ctrl diet. Dietary HM depressed the mRNA level of inflammatory cytokine as compared to NC but not Ctrl group. Collectively, the diet with 300 mg/kg HM has a favorable effect in decreasing the lipid deposition and protecting liver injury by alleviating hepatic oxidant stress and inflammation in post-peak laying hens.

## INTRODUCTION

It is established that the liver is a major part of lipid metabolism and responsible for nearly 95% de novo lipogenesis in birds. However, excessive lipid accumulation can lead to hepatocyte death and further cause liver dysfunction. (Yao et al., 2016). One of classic examples is the fatty liver hemorrhagic syndrome (**FLHS**), which is characterized by increased hepatic triacylglycerol (**TG**) content accompanied by liver hemorrhage (Lee et al., 2010). Additionally, an over many free fatty acid (**FFA**) probably induced the hepatic lipotoxity via activating the tumor necrosis factor alpha (**TNF-α**) expression in mice (Feldstein et al., 2004). All these conditions can hazardously threaten aviculture, compromise animal welfare, and cause significant economic loss.

Multiply nutrient strategies such as choline chloride (Dong et al., 2019), vitamins (Geng et al., 2018), and betaines (Omer et al., 2020) are currently widespread used to relieve hepatic lipid deposition and liver damage in laying hens. Accordingly, Chinese herbs have been receiving a tremendous amount of attention due to its affordability, ready availability, and slight side effect. Accumulating evidence indicated that the liver injury-alleviating effects exerting by Chinese herbs are inseparable from the reduction in fat deposition, oxidative stress, and inflammatory response (Colturato et al., 2012; Mittal et al., 2016). For instance, Andrographolide (**AGL**), major labdane diterpenoid, has been regarded as potent hepatoprotection role in alleviating inflammation both in rodent and cell models (Jin et al., 2011; Shen et al., 2013). AGL was also noticed to regulate the nuclear factor (erythroid-derived-2)-like 2 (**Nrf2**) signaling pathway to ameliorate the acute liver injury induced by d-galactosamine/lipopolysaccharide (Pan et al., 2014). In addition, silymarin, a flavonoid derived from *Silybum marianum*, exhibited strong antioxidant and free radical scavenging abilities through enhancing the production of superoxide dismutase (**SOD**) and glutathione (**GSH**), it has the capabilities of inhibiting lipid peroxidation (Vargas-Mendoza et al., 2014). A study on broilers showed that Chinese herbal could upregulate the expression of peroxisome proliferator activated receptor, the transcription factor sterol regulatory element binding protein-1c (**SREBP-1c**) in liver, and thus decrease plasma TG and nonesterified fatty acid (**NEFA**) concentrations (Xie et al., 2017). Moreover, supplementation of Neem leaf extracts was noticed to decrease the deposition of abdominal fat that is regarded as a production cost in broilers (Paul et al., 2020). Chinese medicine herb composed of *Galla Chinensis, Andrographis paniculata, Arctii Fructus, Glycyrrhizae Radix*, and *Schizonepeta tenuifolia* was observed to improve the average body weight and glutathione peroxidase of broilers (Gao et al., 2022). These data imply that these bioactive ingredients could be a promising strategy for improving the inflammation and antioxidant status as a natural feed additive in laying hens.

In the present study, *Andrographis paniculate, Silybum marianum, Azadirachta Indica*, and *Ocimum basilicum* were selected as the herbaceous mixture (**HM**), and further determined the effect of HM on lipid accumulation of liver and hepatic injury in post-peak layers hens. Furthermore, the relationship between HM and the changes of hepatic function, antioxidant and inflammatory responses was also assessed.

## MATERIALS AND METHODS

All the procedures were conducted in this study under the guidelines of the Animal Health and Care Committee in Henan Agricultural University.

### Preparation of Herbaceous Mixture

The HM was provided by Henan Jinqianguo Bio Tech Co., Ltd. After drying at 40°C for 36 h, *Ocimum basilicum* leaf, *Andrographis paniculata* leaf, *Silybun marianum* fruit, and *Azadirachta indica* leaf were ground to a size that could pass through a 1-mm screen and mixed with glycine as the ratio of 20:35:10:20:15 (Table 1), respectively. Here, the mixing ratio among 4 herbs and the dose of 300 mg/kg HM in diet were chosen based on its minimal concentration of serum alanine aminotransferase (**ALT**) activity, an indicator of liver health, in preliminary trial period (data not shown). All samples were stored at 4°C until use.

**Table 1:** Composition of herbaceous mixture (HM).

Item	Proportion (%)
*Ocimum basilicum*	20
*Andrographis paniculata*	35
*Silybun marianum*	10
*Azadirachta indica*	20
Glycine	15

### Birds, Diets, and Management

A total of 240 health 389-day-old Hy-line Brown hens were randomly divided into 3 treatment groups with 8 replicates (30 birds/replicate) for 28 d, including 1) the negative control group (**NC**; feeding the no choline chloride basal diet), 2) the choline chloride control group (**Ctrl**; feeding the basal diet with 1,000 mg/kg choline chloride based on the practice), and 3) the HM group (HM; feeding the basal diet with 300 mg/kg HM). All hens were housed in stainless steel cages in a climate-controlled facility. The temperature in the room was 24 ± 2°C with humidity 40 to 60% based on normal management practices. The light schedule was 16L:8D throughout the trial. The basal diet and nutritional compositions were listed in Table 2 according to China Agricultural Industry Standards (NY/T 33-2004). The diet was supplied in the form of powder, and all birds were given ad libitum access to feed and water.

**Table 2:** Composition and calculated nutrient content (as-fed).

Items	No choline chloride basal diet
Ingredients, %	
Corn grain	33.65
Soybean meal, 43% Crude protein	6.50
Soybean meal, 46% Crude protein	13.65
Corn gluten powder	16.50
Corn gluten meal	1.50
Wheat middling	24.00
Limestone	1.53
Monocalcium phosphate	1.20
DL-Methionine	0.15
L-Lysine HCl	0.30
Premix 1	1.02
Total	100.0
Calculated nutrient composition, %	
Metabolizable energy, MJ/kg	11.28
Crude protein	18.50
Calcium	1.00
Total phosphorus	0.60
Available phosphorus	0.42
Lysine	0.87
Methionine + Cysteine	0.68

1 Premix providing per kg of diet: vitamin A (retinyl acetate), 2,700 IU; vitamin D_3_ (cholecalciferol), 3,400 IU; vitamin E (dl-α-tocopherol acetate), 10 mg; vitamin K_3_ (menadione), 0.5 mg; vitamin B_1_ (thiamine), 2.0 mg; vitamin B_2_ (riboflavin), 5 mg; niacin, 30 mg; D-pantothenic acid, 10 mg; vitamin B_6_ (pyridoxine-HCl), 3 mg; vitamin B_12_ (cyanocobalamine), 7 µg; folic acid, 0.5 mg; biotin, 0.1 mg; Fe (FeSO_4_.H_2_O), 80 mg; Cu (CuSO_4_.5H_2_O), 8 mg; Zn (ZnO), 80 mg; Mn (MnSO_4_), 80 mg; I (KI), 0.7 mg; Se (Na_2_O_3_Se), 0.3 mg.

### Productive Performance and Sample Collection

The egg numbers, egg weight, and feed intake were recorded daily. Egg production, average daily feed intake, and feed conversion ratio expressed as the ratio of grams of total feed intake to grams of total egg weight was calculated. Upon completing this experiment, 8 birds (1 bird per cage) were selected and weighted from each treatment, fasting for 12 h, for blood sampling via the jugular vein. Blood was centrifuged at 3,500 rpm/15 min at 4°C, and serum was prepared for further analysis. Subsequently, these layers were sacrificed through cervical dislocation, and the abdominal fat and liver were weighted. The liver samples were obtained and immediately snap-frozen in liquid nitrogen and stored at −80°C for determination of hepatic lipid metabolism, antioxidant activity, and inflammatory reaction. A part of liver issue was fixed with 4% formaldehyde for morphological analysis.

### Serum Lipid Profile

Serum TG, total cholesterol (**TC**), high-density lipoprotein cholesterol (**HDL-c**), and low-density lipoprotein cholesterol (**LDL-c**) were qualitied using an automatic biochemistry analyzer (YSI Incorporated, Yellow Springs, OH\).

### Hepatic Lipid Accumulation

Hepatic TG, TC, FFA were measured with commercial assay kits (Nanjing Jiancheng Bioengineering Institute, Nanjing, China) as described by Zhang et al. (2020). In detail, the liver was diluted by saline as 1 g: 9 mL for grinding. The liver grinding fluid was centrifugated at 12,000 rpm/10 min at 4°C, and the supernatant was collected for determination of protein, TG, TC, and FFA. All measurements of each variable were runed in the same assay to avoid intra-assay variability. Results of parameters were calculated as millimole per gram protein (mmol/g protein). Moreover, formalin-fixed liver samples were dehydrated, embedded, sliced into 5-μm transects, and stained with Oil red O dying for visualizing hepatic lipid deposition (Wang and Ching, 2021).

### Assessment of Liver Injury

Fixed liver samples were processed with paraffin-embedding, and then cut into 6 μm. Hematoxylin and eosin (**H&E**) staining was performed using standard procedures. The images were obtained by using an inverted microscope and CellSens standard processing system (PLYMPUS). The area and diameter of hepatic vacuoles were quantified by Image-Pro Plus (IPP 6.0, Cyber Medianetics). The distribution histogram of lipid droplet diameter was determined by Origin Pro (2021b, Origin Lab, Northampton, MA). Moreover, serum AST were also examined using a diagnostic kit provided by Thermo Scientific (Waltham, MA).

### Measurements of Hepatic Lipid Peroxidation

The hepatic total antioxidative capacity (**T-AOC**), SOD, glutathione peroxidase (**GSH-Px**), catalase (**CAT**), malondialdehyde (**MDA**), and protein were performed using commercial kits (Nanjing Jiancheng Bioengineering Institute Co. Ltd) according to specification. Results were defined as units per milligram protein (mg protein). All samples were tested for triplicate within each assay.

### Gene Expression Assays

Hepatic total RNA was obtained by Trizol reagent (TransGen Biotech Co. Ltd, Beijing, China). After checking purity and concentration, the cDNA was reverse transcript from total RNA by commercial kit (Vazyme Biotech Co. Ltd, Nanjing, China). The obtained cDNA was used to determine the mRNA expression of genes involved in hepatic lipid metabolism, hepatocytes apoptosis, and inflammatory cytokines using ABI 7900 real-time PCR system with SYBR qPCR Master Mix (Vazyme Biotech Co. Ltd). Target cDNA was amplified by 40 cycles (1 cycle: 95°C/30 s, annealing at 60°C/60 s) and a final melting curve analysis. A standard curve was generated to estimated reaction efficiency (slope) and genes expression. Primers were designed using online Primer 3 and are shown in Table 3. *β-actin* was used to normalize the relative amounts of RNAs of interest.

**Table 3:** The primers for quantitative real-time PCR.

Gene name	Gene ID	Primer (5’-3’)	Product size (bp)
*FAS*	NM_205155.3	F: TGCTATGCTTGCCAACAGGA R: ACTGTCCGTGACGAATTGCT	128
*ACC-1*	NM_205505.1	F: AGTGGATAACTGCTCAGATTGC R: AGGGTTCATCTCCAGGGGTT	106
*SREBP-1*	NM_204126.2	F: CTGGCTGAAGGGTGACGAG R: CCGTCCTGCTTGCTCAACAT	150
*HSL*	XM_025155301.1	F: TGTTTGGCTCAGGGGGTTTT R: ATGGATGGCACGAACTGGAA	155
*LPL*	NM_205282.1	F: TGTTTGGCTCAGGGGGTTTT R: ATGGATGGCACGAACTGGAA	187
*SCD*	NM_204890.1	F: AGCAGAACGAGGCATGGTAG R: GAGCACTCAACACGAAGCAC	203
*MTTP*	NM_001109784.2	F: GGGCAGTCCAGCATGATTGT R: CAGGGATGCTTTAGCCCGAT	165
*LDLR*	NM_204452.1	F: CTGTGAGGGCCTTTGTCTGC R: GGCCGTGGTGGAGTTGG	157
*VDLR*	NM_205229.1	F: GTGGTCAGTGTGTGCCGAA R: GGACACTGGGATACACTGGGT	152
*TNF-α*	NM_204267.1	F: GAGCAGGGCTGACACGG R: GCTGCTTCCAAATGCCTCAG	79
*IL-6*	NM_204628.1	F: ACTCGTCCGGAGAGGTTGG R: TCTCCATGCTGTTCTCGCAC	153
*Bcl-2*	NM_205339.2	F: ACCTGGATGACCGAGTACCT R: GGCCTCATACTGTTGCCGT	101
*Beclin-1*	NM_001006332.1	F: AGACCAGCTAGACACACAGC R: TCAGTTCTGTTTGCAGTTTCTCC	119
*Caspase 3*	NM_204725.1	F: TCTGCCTGATGACAGTTACAGA R: CATCTGCATCCGTGCCTGAA	119
*Caspase 8*	*NM_204592.3*	F: TGGAGAGAGAGCTCCAGGTT R: CTGACAGCTGAAAGAGCAGA	72
*β-actin*	NM_205518.1	F: CTGTGTTCCCATCTATCGT R: TCTTCTCTCTGTTGGCTTTG	270

Abbreviations: ACC-1, acetyl-CoA carboxylase 1; Bcl-2, B-cell leukemia/ lymphoma 2; FAS, fatty acid synthase; HSL, hormone-sensitive lipase; IL, interleukin-6; LPL, lipoprotein lipase; LDLR, low-density lipoprotein receptor; MTTP, microsomal triglyceride transfer protein SREBP-1, sterol regulatory element binding protein 1; SCD, and stearoyl-CoA desaturase; TNF-α, tumor necrosis factor-α; VLDLR, very low-density lipoprotein receptor.

### Statistical Analysis

Results were presented as means ± standard error (**SE**). The Shapiro-Wilk and Brown-Forsythe test methods were used to test the normality and homogeneity of data, respectively. The data were analyzed using one way-analysis of variance (**ANOVA**) followed by Tukey multiple comparison tests by SPSS (Version 23 IBM, Armonk, NY). *P* < 0.05 was regarded as difference significantly.

## RESULTS

### Reproduction Performance

**Figure 1:**
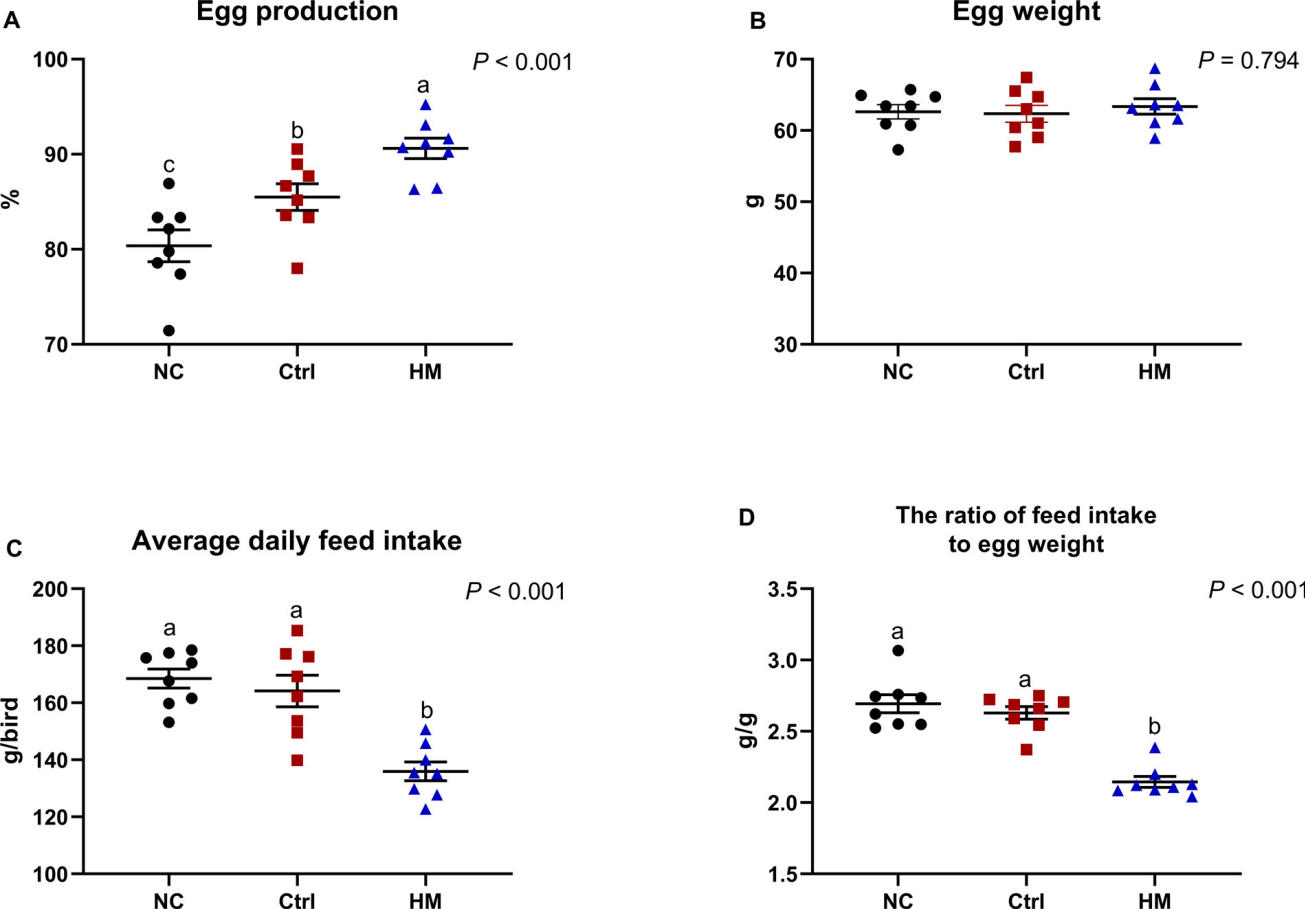
The effect of dietary herbaceous mixture (HM) supplementation on the production performance of laying hens including (A) egg production, (B) egg weight, (C) average daily feed intake, and feed conversion ration expressed as the ratio of grams of total egg weight to grams of total feed intake. Values are presented as mean ± standard error (n = 6). Different letters in the same column represent the significant difference (*P* < 0.05). Abbreviations: Ctrl, choline chloride control group; NC, negative control.

At presented in Figure 1, compared with the NC group, both the Ctrl and HM group significantly increased the egg production (Figure 1A). All treatment groups had no significant effect on the average egg weight during the overall of experiment period (*P* > 0.05; Figure 1A). A significantly decreased in ADFI was observed in the HM group as compared to NC or Ctrl group (Figure 1C), and thereby increased the feed intake to egg weigh ratio (*P* < 0.001; Figure 1D). Overall, our experiment indicated that dietary supplementation HM can improve the laying performance of laying hens.

### HM Decreased the Relative Abdominal Fat Weight With Similar BW

As shown in Table 4, there was no difference in BW, absolute abdominal fat weight, absolute liver weight, and relative liver weight among NC, Ctrl, and HM groups (*P* > 0.05). However, the HM decreased the weight of abdominal fat normalized to BW when compared with NC group (*P* > 0.05) and Ctrl group (*P* < 0.05).

**Table 4:** Effect of herbaceous mixture on body weight and organ size of post-peak laying hens.

Items	NC	Ctrl	HM	*P-*value
Body weight, kg	1.98 ± 0.16	2.02 ± 0.29	2.05 ± 0.10	0.231
Abdominal fat weight, g	86.87 ± 28.05	90.47 ± 38.09	63.51 ± 15.84	0.118
Relative abdominal fat weight, %	4.51 ± 1.23^ab^	5.33 ± 1.96^a^	3.35 ± 0.75^b^	0.008
Liver weight, g	32.65 ± 6.24	30.94 ± 6.63	30.38 ± 6.71	0.790
Relative liver weight, %	1.72 ± 0.27	1.46 ± 0.21	1.54 ± 0.31	0.238

Values are mean ± standard error (n = 8).

Abbreviations: Ctrl, choline chloride control group; HM, herbaceous mixture; NC, negative control.

ab Different letters in the same column represent the significant difference (*P* < 0.05).

### HM Improved Serum Lipid Profile

**Figure 2:**
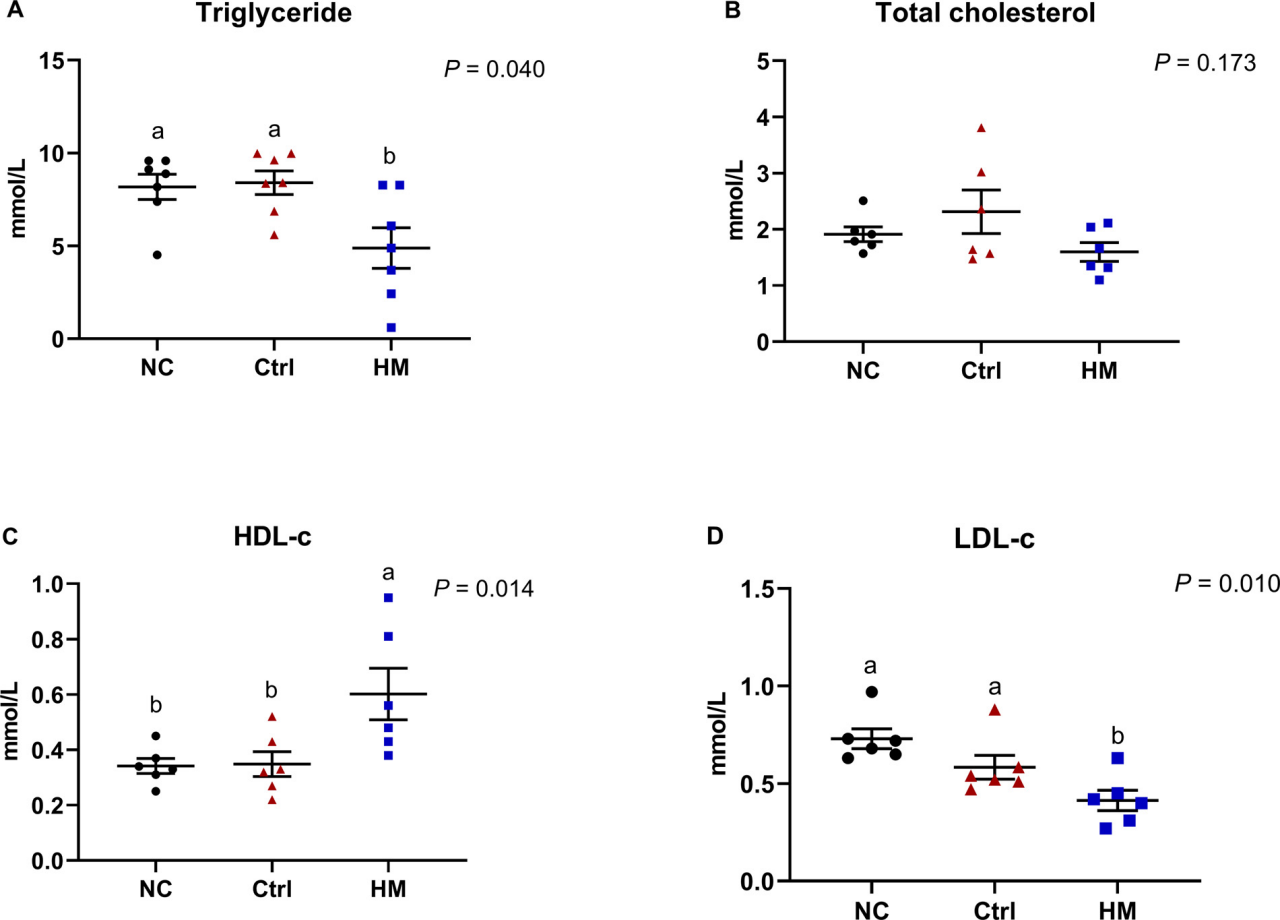
Herbaceous mixture (HM) improved serum lipid profile in post-peak laying hens. The concentration of (A) triglyceride, (B) total cholesterol, (C) high density lipoprotein cholesterol (HDL-c), and (D) low density lipoprotein cholesterol (LDL-c) in serum. Values are presented as mean ± standard error (n = 6). Different letters in the same column represent the significant difference (*P* < 0.05). Abbreviations: Ctrl, choline chloride control group; NC, negative control.

The effects of HM on serum lipid profile were illustrated in Figure 2. When compared with both NC and Ctrl group, birds fed the diet supplemented with HM exhibited a significant (*P* = 0.040) decrease in serum TG concentration (Figure 2A), but no significant difference regarding serum TC levels (*P* > 0.05, Figure 2B). As far as lipoprotein concerned, the diet with HM possessed higher HDL-c (*P* = 0.014) and lower LDL-c levels (*P* = 0.010) relative to NC and Ctrl groups (Figures 2C and 2D)

### HM Alleviated Hepatic Lipid Deposition via Inducing Lipolysis

**Figure 3:**
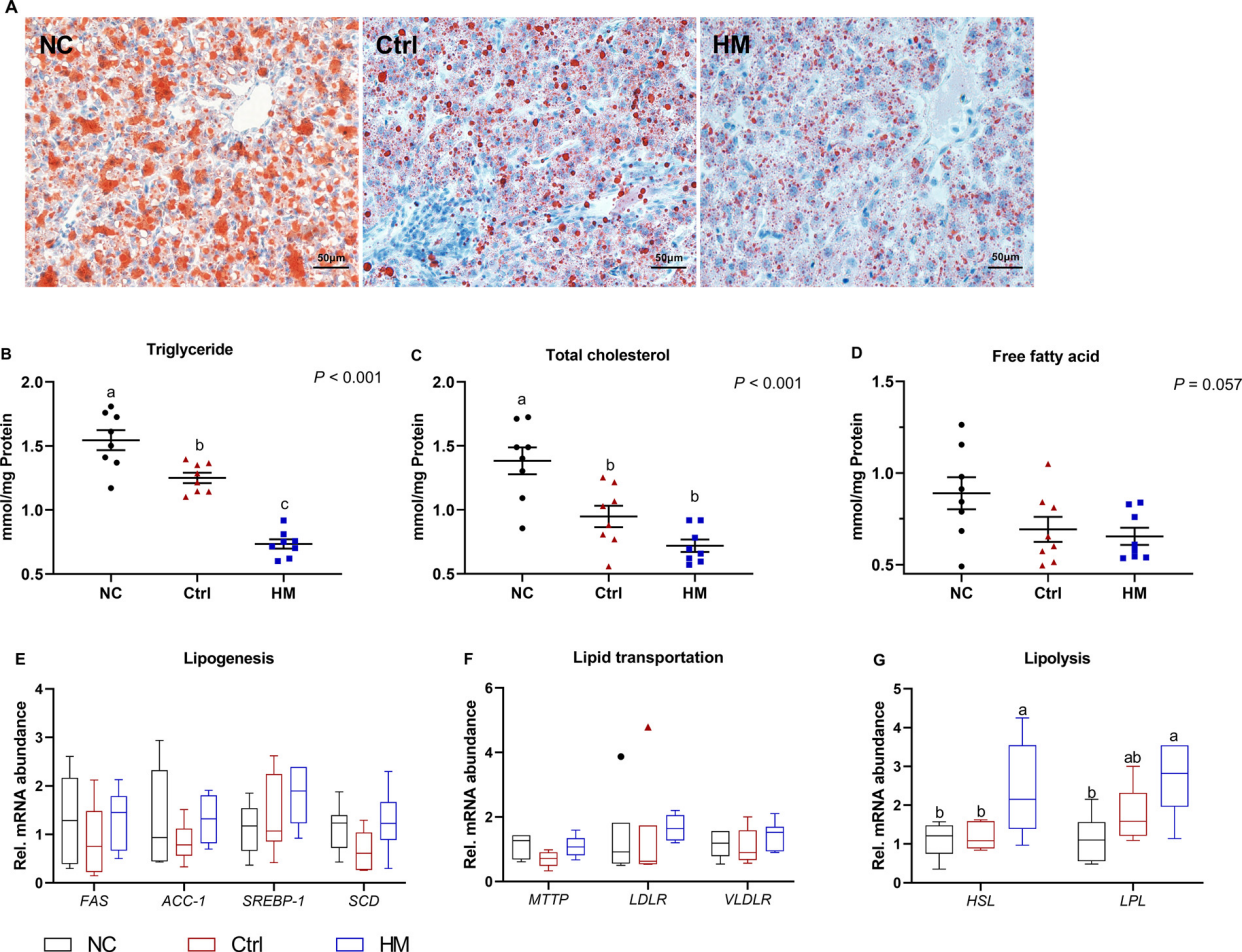
Hepatic lipid metabolism response to dietary herbaceous mixture (HM) treatment in post-peak laying hens. (A) Liver oil red O staining (scale: 50 μm), the concentration of (B) triglyceride, (C) total cholesterol, and (D) free fatty acid (FFA) of liver (n = 8). mRNA abundance of (E) lipogenesis including fatty acid synthase (*FAS*), acetyl CoA carboxylase 1 (*ACC-1*), sterol regulatory element binding transcription factor 1 (*SREBP-1*), and stearoyl-CoA desaturase (*SCD*), (F) lipid transportation, i.e., microsomal triglyceride transfer protein (*MTTP*), low density lipoprotein receptor (*LDLR*), and very low-density lipoprotein receptor (*VLDLR*), and (G) lipolysis including hormone-sensitive lipase (*HSL*) and lipoprotein lipase (*LPL*) (n = 6). Values are presented as mean ± standard error. Different letters in the same column represent the significant difference (*P* < 0.05). Abbreviations: Ctrl, choline chloride control group; NC, negative control.

To investigate whether HM could reduce the hepatic accumulation of lipid in post-peak laying hens. The outcome of the Oil red O staining showed that the lipid accumulation in liver was decreased by Ctrl or HM diet compared to NC group (Figure 3A), which was further supported by the decreased the TG and TC levels in liver. That is, the diets supplemented of HM resulted to lower level of TG and TC (*P* < 0.001), as well as tended to decline (*P* = 0.057) the level of FFA relative to NC group (Figures 3B–3D).

Concerned the expressions of lipid metabolism-related genes. The genes involving in fatty acid synthesis including fatty acid synthase (***FAS***), acetyl-CoA carboxylase 1 (***ACC-1***), *SREBP-1*, and stearoyl-CoA desaturase (***SCD***) were comparable among NC, Ctrl, and HM groups (*P* > 0.05, Figure 3E). Analogously, the lipid transportation gene, microsomal triglyceride transfer protein (***MTTP***), low-density lipoprotein receptor (***LDLR***), and very low-density lipoprotein receptor (***VLDLR***) was not significantly changed by experimental treatment (*P* > 0.05, Figure 3F). Regarding fatty acid β-oxidation, the diet with HM notably upregulated the expression of hormone-sensitive lipase (***HSL***) and lipoprotein lipase (***LPL***) when compared with NC and Ctrl group (both *P* < 0.05, Figure 3G).

### HM Attenuated Liver Steatosis and Hepatocyte Apoptosis

**Figure 4:**
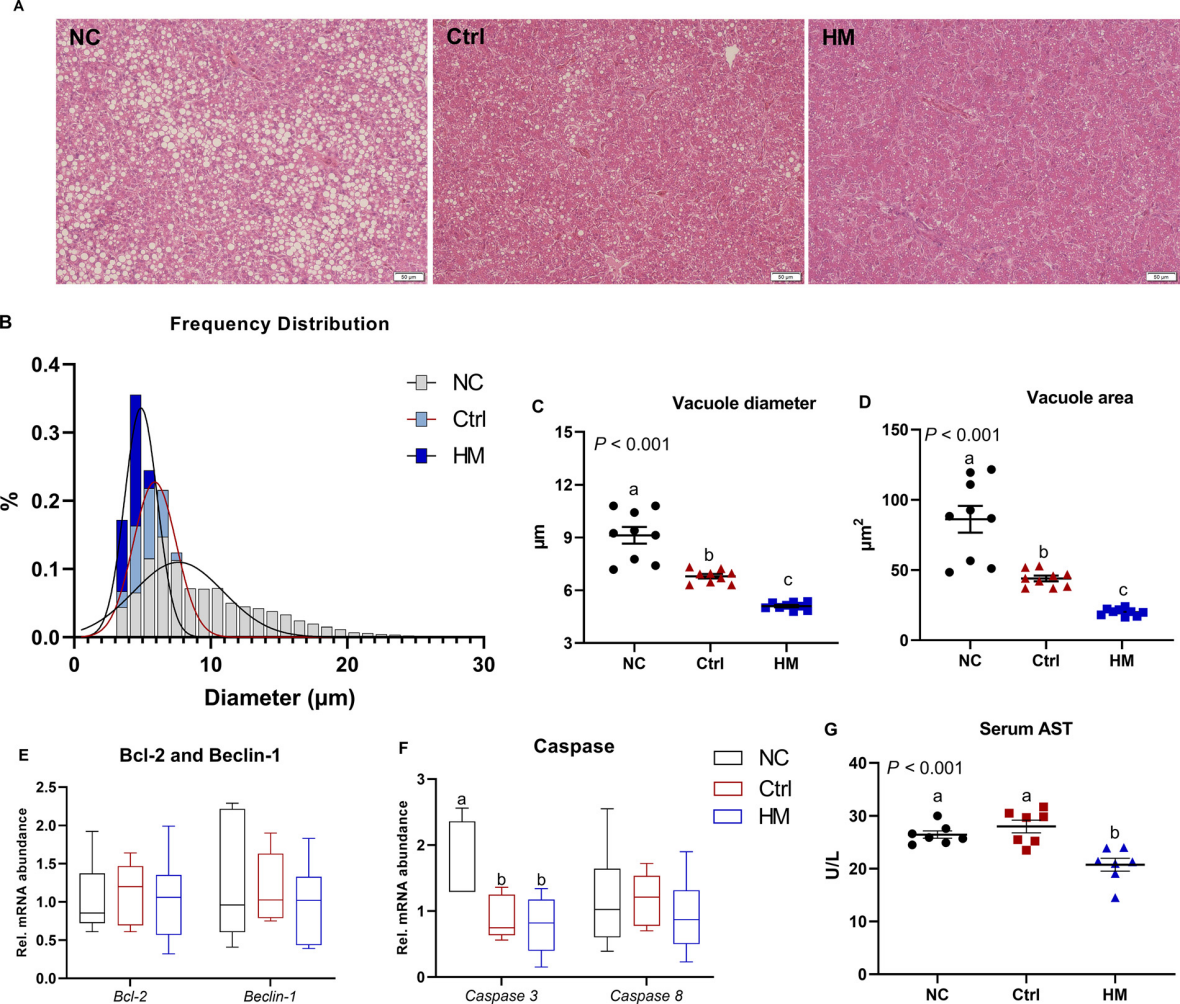
Herbaceous mixture (HM) attenuated liver steatosis and hepatocyte apoptosis in post-peak laying hens. (A) Liver hematoxylin and eosin (H&E) staining (scale: 50 μm) was performed, and (B) frequency distribution of vacuole, (C) the diameter and (D) area were quantified based on the H&E section (n = 8). The mRNA expression of genes involved in apoptosis including (E, F) *Beclin-1*, B-cell leukemia/ lymphoma 2 (*Bcl-2*), *Caspase 3*, and *Caspase 8* were measured by RT-PCR (n = 6). (G) Serum aspartate transaminase (AST) was determined (n = 7). Values are presented as mean ± standard error. Different letters in the figure represent the significant difference (*P* < 0.05). Abbreviations: Ctrl, choline chloride control group; NC, negative control.

To further study the hepatic pathological status, liver was subjected to H&E staining and the diameter and area of vacuole were measured. As indicated in Figure 4A, the most remarkable alteration was the formation of vacuoles in the liver of NC birds (Figure 4B). Quantitative analysis of bubbles indicated that the NC group exhibited a higher mean vacuole diameter, whereas Ctrl and HM administration decreased the bubbles’ diameter and area (Figure 4C, D; both *P* < 0.001). The outcomes of cell apoptosis revealed that Ctrl and HM diet notably downregulated the mRNA abundance of *Caspase 3* (*P* < 0.01), but not apparently changed the transcription of *Beclin-1*, B-cell leukemia/ lymphoma 2 (***Bcl-2***), and *Caspase 8* (Figures 4E and 4F). Moreover, the serum liver injury marker, AST activity, was notably decreased by HM treatment as compared with both NC and Ctrl group (Figure 4G).

### HM Improved Hepatic Antioxidant and Anti-inflammation Status

**Figure 5:**
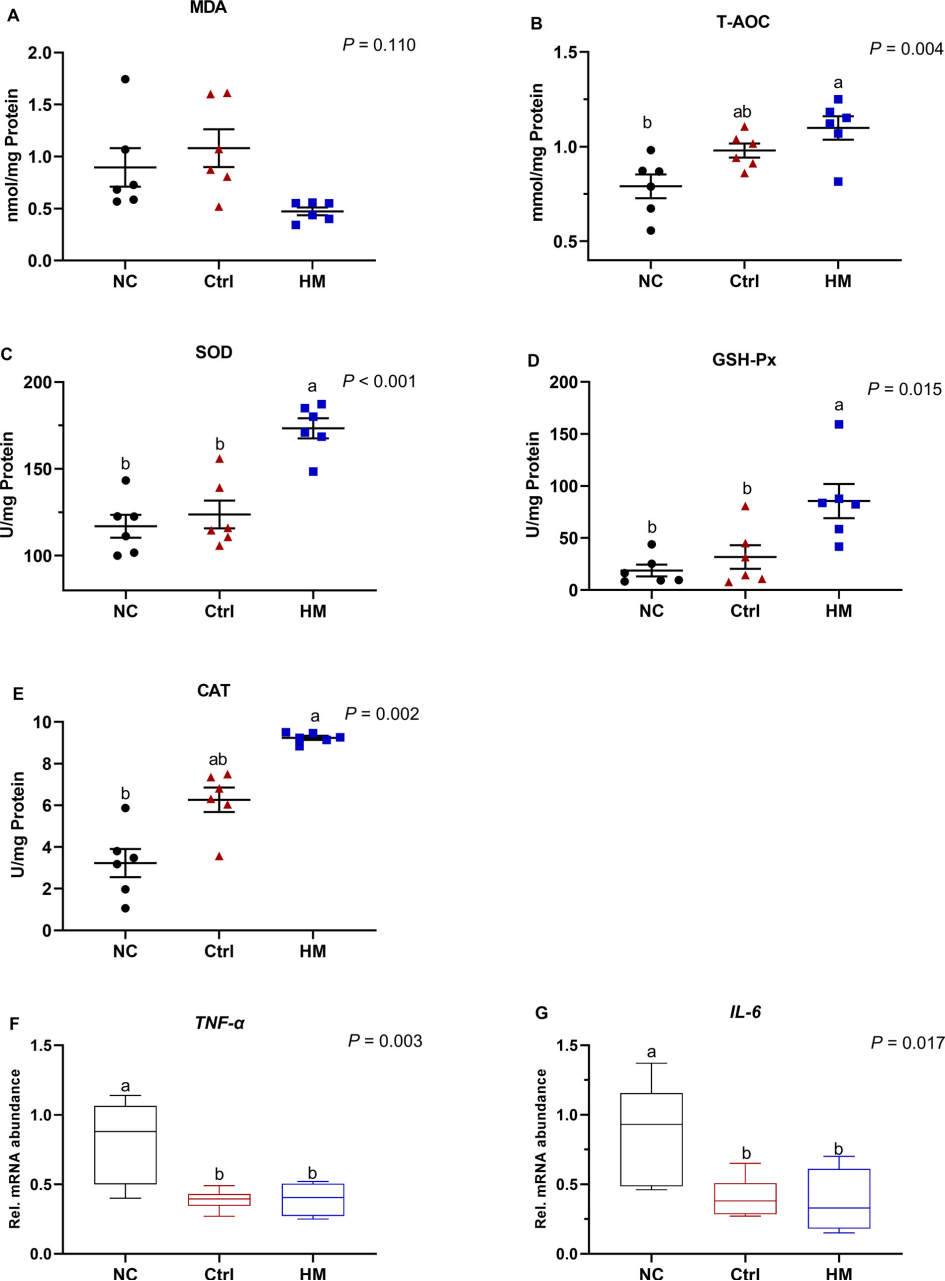
Effects of herbaceous mixture (HM) on hepatic oxidative stress and inflammation in post-peak laying hens. The antioxidative stress was evaluated through (A) malondialdehyde (MDA), (B) total antioxidative capacity (T-AOC), (C) superoxide dismutase (SOD), (D) glutathione peroxidase (GSH-Px), and (E) catalase (CAT); RT-PCR analysis of proinflammatory cytokines including (F) tumor necrosis factor-α (*TNF-α*) and (G) *interleukin-6*. Values are presented as mean ± standard error (n = 6). Different letters in the same column represent the significant difference (*P* < 0.05). Abbreviations: Ctrl, choline chloride control group; NC, negative control.

We next explored the mechanism underlying HM alleviating liver injury of laying hens. As showed in Figure 5A, no significant alteration was found in MDA level among these groups (*P* = 0.110), dietary HM treatment enhanced the activities of antioxidant enzymes in the liver, indicated by the higher activity of SOD, GSH-Px, CAT, and T-AOC, when compared to NC group (Figures 5B–5E; *P* < 0.05). Determining hepatic expression of inflammatory genes found that both HM and Ctrl diets significantly downregulated the mRNA expression of *TNF-α* and *IL-6* mRNA in the liver as compared with those in the NC diets (Figures 5F–5G; *P* < 0.05).

## DISCUSSION

FLHS is one of widespread metabolic diseases in laying hens, which is mainly related to diet nutritional composition and excessive feed intake (Yang et al., 2017). Chinese herbs have been noticed to display beneficial effect on reduction of lipid metabolism in poultry (Sugiharto et al., 2020). In the present study, we revealed that the diet with 300 mg/kg HM increased the egg production accompanied by decreased abdominal fat mass in hens. Furthermore, the diet with 300 mg/kg HM has a protective effect on hepatic health of post-peak laying hens through attenuating lipid accumulation, oxidative stress, and inflammation of liver.

Several lines of evidence show that Chinese herbs including *Silybum marianum, Azadirachta* improved lipid metabolism in patients (Di Pierro et al., 2018) and broiler chickens (Mafouo Sonhafouo et al., 2019), which was probably associated with higher content of polyphenols and flavones included in HM (Drewnowski, 2001). In the current study, the diet with 300 mg/kg HM improved serum lipid profiled and reduced hepatic lipid accumulation in post-peak laying hens, evidenced by Oil-red O staining and decreased TG, TC, and FFA content in liver, which agreed with previous study in rat saying that herbs extract (*Astragalus membranaceus, Salvia miltiorrhiza*, and *Pueraria lobata*) decreased the hepatic TG and TC content (Jeong et al., 2004). To investigate the mechanism underlying the HM diet decreasing hepatic lipid deposition, the mRNA expression of genes involved in lipid metabolism was further analyzed and revealed the HM diet declined hepatic lipid accumulation was unrelated to lipogenesis and transportation. That is, HM did not change the transcription of lipogenesis genes (*FAS, ACC1, SREBP1*, and *SCD*) and transportation genes (*MTTP, LDLR*, and *VLDLR*). However, the upregulation in *HSL* and *LPL* mRNA expression induced by the HM diet may lead to the increase in intracellular cyclic adenosine monophosphate levels and activation in the dormant protein kinase A, which in turn to increase lipolysis (Lampidonis et al., 2011), indicating that HM could promote lipolysis and consequently alleviate hepatic lipid deposition. Consistent with our observations, downregulated the lipogenesis gene to decrease fat masses and serum TG and LDL-c level also noticed in tea polyphenols-treated broilers (Huang et al., 2013).

It was reported that hepatocytes were vulnerable to FFA-induced oxidative stress and inflammation when exposed to high levels of circulating FFAs, and thus further lead to hepatocyte apoptosis (Dowman et al., 2010). In this study, the birds fed no choline chloride basal diets exhibited a higher vacuole diameter and area, as well as increased AST activity and *Caspase 3* expression, which were reversed by dietary HM treatment, implying that HM attenuated steatosis and apoptosis of liver in post-peak laying hens. One of important mechanisms underlying the lessening of liver injury by dietary HM probably involved in the improved antioxidative status. In the present study, in addition to decreased MDA content, the activities of T-AOC, SOD, GSH-Px, and CAT in liver were increased significantly by the HM administration in layer's liver. Moreover, regardless used in combination or single, Chinese herbs were found to play a key role in the development of hepatic antioxidative defense (Yan et al., 2020). According to the study on hyperlipidemia rats, 1 L of aqueous *Ocimum basilicum* extract possessed the homologous antioxidant capacity as 32.8 g of ascorbic acid, which indicated the powerful potential of Chinese herbs in antioxidation system (Amrani et al., 2006). An obvious restoration of antioxidation system was also observed in silymarin-pre-treated rats, which companied by a decrease in lipid peroxidation (Singh et al., 2012). In broiler, Chinese medicine herbs were similarly noticed to improve the average body weight and glutathione peroxidase (Gao et al., 2022).

Additional possible mediators of HM alleviating liver injury probably are associated with the decreased hepatic inflammation. A tight relationship between hepatic disease and inflammation has been identified, which partly depends on the activation of nuclear factor kappa-B (**NF-κB**) signaling pathway (Sunami et al., 2012). Emerging evidence indicated that Chinese herbs have a potent anti-inflammatory action throughout the suppression of NF-κB regulated gene expression, especially inflammatory cytokines (Bannwart et al., 2010). Sweet basil extracts could reduce the mRNA level of *NF-κB* and inflammatory cytokine including *IL-6, IL-1β* and *TNF-α* in co-culture of 3T3-L1 adipocytes and RAW264.7 macrophages (Takeuchi et al., 2020). More important, the liver lesions induced by thioacetamide in male mice could be attenuated by silymarin due to its lower hepatic inflammation reaction (Chen et al., 2012). In this study, the HM diet downregulated the mRNA expression of *IL-1β* and *IL-6* mRNA in the liver, indicating that decreasing inflammatory cytokines due to dietary HM manipulation might be protentional contributor to alleviate hepatocytes apoptosis.

In summary, the diet with 300 mg/kg HM exerted a beneficial effect on lipid metabolism and improved hepatic health status in post-peak laying hens. Specifically, dietary HM supplementation reduced hepatic lipid deposition, liver steatosis, and hepatocyte apoptosis by improving hepatic antioxidant and anti-inflammation status in layers.

## References

[bib0001] Amrani S., Harnafi H., Bouanani Nel H., Aziz M., Caid H.S., Manfredini S., Besco E., Napolitano M., Bravo E. (2006). Hypolipidaemic activity of aqueous Ocimum basilicum extract in acute hyperlipidaemia induced by triton WR-1339 in rats and its antioxidant property. Phytother Res..

[bib0002] Bannwart C.F., Nakaira-Takahagi E., Golim M.A., de Medeiros L.T., Romao M., Weel I.C., Peracoli M.T. (2010). Downregulation of nuclear factor-kappa B (NF-kappaB) pathway by silibinin in human monocytes challenged with Paracoccidioides brasiliensis. Life Sci..

[bib0003] Chen I.S., Chen Y.C., Chou C.H., Chuang R.F., Sheen L.Y., Chiu C.H. (2012). Hepatoprotection of silymarin against thioacetamide-induced chronic liver fibrosis. J. Sci. Food Agric..

[bib0004] Colturato C.P., Constantin R.P., Maeda A.S., Constantin R.P., Yamamoto N.S., Bracht A., Ishii-Iwamoto E.L., Constantin J. (2012). Metabolic effects of silibinin in the rat liver. Chem. Biol. Interact..

[bib0005] Di Pierro F., Putignano P., Villanova N. (2018). Retrospective analysis of the effects of a highly standardized mixture of Berberis aristata, Silybum marianum, and monacolins K and KA in diabetic patients with dyslipidemia. Acta Bio-med.: Atenei Parmensis.

[bib0006] Dong X.F., Zhai Q.H., Tong J.M. (2019). Dietary choline supplementation regulated lipid profiles of egg yolk, blood, and liver and improved hepatic redox status in laying hens. Poult. Sci..

[bib0007] Dowman J.K., Tomlinson J.W., Newsome P.N. (2010). Pathogenesis of non-alcoholic fatty liver disease. QJM.

[bib0008] Drewnowski A. (2001). The science and complexity of bitter taste. Nutr. Rev..

[bib0009] Feldstein A.E., Werneburg N.W., Canbay A., Guicciardi M.E., Bronk S.F., Rydzewski R., Burgart L.J., Gores G.J. (2004). Free fatty acids promote hepatic lipotoxicity by stimulating TNF-alpha expression via a lysosomal pathway. Hepatology.

[bib0010] Gao J., Wang R., Liu J., Wang W., Chen Y., Cai W. (2022). Effects of novel microecologics combined with traditional Chinese medicine and probiotics on growth performance and health of broilers. Poult. Sci..

[bib0011] Geng Y., Ma Q., Wang Z., Guo Y. (2018). Dietary vitamin D3 supplementation protects laying hens against lipopolysaccharide-induced immunological stress. Nutr. Metab. (Lond).

[bib0012] Huang J., Zhang Y., Zhou Y., Zhang Z., Xie Z., Zhang J., Wan X. (2013). Green tea polyphenols alleviate obesity in broiler chickens through the regulation of lipid-metabolism-related genes and transcription factor expression. J. Agric. Food Chem..

[bib0013] Jeong K.H., Yun Y.K., Young C.S. (2004). Effects of natural product extract on the fatty liver induced by alcohol diet in rats. J. Health Sci..

[bib0014] Jin L., Shi G., Ning G., Li X., Zhang Z. (2011). Andrographolide attenuates tumor necrosis factor-alpha-induced insulin resistance in 3T3-L1 adipocytes. Mol. Cell Endocrinol..

[bib0015] Lampidonis A.D., Rogdakis E., Voutsinas G.E., Stravopodis D.J. (2011). The resurgence of hormone-sensitive lipase (HSL) in mammalian lipolysis. Gene.

[bib0016] Lee B.K., Kim J.S., Ahn H.J., Hwang J.H., Kim J.M., Lee H.T., An B.K., Kang C.W. (2010). Changes in hepatic lipid parameters and hepatic messenger ribonucleic acid expression following estradiol administration in laying hens (Gallus domesticus). Poult. Sci..

[bib0017] Mafouo Sonhafouo V., Kana J.R., Nguepi Dongmo K. (2019). Effects of graded levels of Azadirachta indica seed oil on growth performance and biochemical profiles of broiler chickens. Vet. Med. Sci..

[bib0018] Mittal S.P.K., Khole S., Jagadish N., Ghosh D., Gadgil V., Sinkar V., Ghaskadbi S.S. (2016). Andrographolide protects liver cells from H2O2 induced cell death by upregulation of Nrf-2/HO-1 mediated via adenosine A2a receptor signalling. Biochim. Biophys. Acta..

[bib0019] Omer N.A., Hu Y., Idriss A.A., Abobaker H., Hou Z., Yang S., Ma W., Zhao R. (2020). Dietary betaine improves egg-laying rate in hens through hypomethylation and glucocorticoid receptor-mediated activation of hepatic lipogenesis-related genes. Poult. Sci..

[bib0020] Pan Y.E., Liu Z.C., Chang C.J., Huang Y.F., Lai C.Y., Walzem R.L., Chen S.E. (2014). Feed restriction ameliorates metabolic dysregulation and improves reproductive performance of meat-type country chickens. Anim. Reprod. Sci..

[bib0021] Paul T.K., Hasan M.M., Haque M.A., Talukder S., Sarker Y.A., Sikder M.H., Khan M., Sakib M.N., Kumar A. (2020). Dietary supplementation of Neem (Azadirachta indica) leaf extracts improved growth performance and reduced production cost in broilers. Vet. World..

[bib0022] Shen T., Yang W.S., Yi Y.S., Sung G.H., Rhee M.H., Poo H., Kim M.Y., Kim K.W., Kim J.H., Cho J.Y. (2013). AP-1/IRF-3 targeted anti-inflammatory activity of andrographolide isolated from Andrographis paniculata. Evid. Based Complement Alternat. Med..

[bib0023] Singh K., Singh N., Chandy A., Manigauha A. (2012). In vivo antioxidant and hepatoprotective activity of methanolic extracts of Daucus carota seeds in experimental animals. Asian Pac. J. Trop. Biomed..

[bib0024] Sugiharto S., Pratama A.R., Yudiarti T., Wahyuni H.I., Widiastuti E., Sartono T.A. (2020). Effect of acidified turmeric and/or black pepper on growth performance and meat quality of broiler chickens. Int. J. Vet. Sci. Med..

[bib0025] Sunami Y., Leithauser F., Gul S., Fiedler K., Guldiken N., Espenlaub S., Holzmann K.H., Hipp N., Sindrilaru A., Luedde T., Baumann B., Wissel S., Kreppel F., Schneider M., Scharffetter-Kochanek K., Kochanek S., Strnad P., Wirth T. (2012). Hepatic activation of IKK/NFkappaB signaling induces liver fibrosis via macrophage-mediated chronic inflammation. Hepatology..

[bib0026] Takeuchi H., Takahashi-Muto C., Nagase M., Kassai M., Tanaka-Yachi R., Kiyose C. (2020). Anti-inflammatory effects of extracts of sweet basil (Ocimum basilicum L.) on a co-culture of 3T3-L1 adipocytes and RAW264.7 Macrophages. J. Oleo Sci..

[bib0027] Vargas-Mendoza N., Madrigal-Santillan E., Morales-Gonzalez A., Esquivel-Soto J., Esquivel-Chirino C., Garcia-Luna Y.G.-R.M., Gayosso-de-Lucio J.A., Morales-Gonzalez J.A. (2014). Hepatoprotective effect of silymarin. World J. Hepatol..

[bib0028] Wang F.Y., Ching T.T. (2021). Oil red O staining for lipid content in caenorhabditis elegans. Bio. Protoc..

[bib0029] Xie Z., Zhang J., Ma S., Huang X., Huang Y. (2017). Effect of Chinese herbal medicine treatment on plasma lipid profile and hepatic lipid metabolism in Hetian broiler. Poult. Sci..

[bib0030] Yan T., Yan N., Wang P., Xia Y., Hao H., Wang G., Gonzalez F.J. (2020). Herbal drug discovery for the treatment of nonalcoholic fatty liver disease. Acta Pharm. Sinica B..

[bib0031] Yang F., Ruan J., Wang T., Luo J., Cao H., Song Y., Huang J., Hu G. (2017). Improving effect of dietary soybean phospholipids supplement on hepatic and serum indexes relevant to fatty liver hemorrhagic syndrome in laying hens. Anim. Sci. J.

[bib0032] Yao X.M., Li Y., Li H.W., Cheng X.Y., Lin A.B., Qu J.G. (2016). Bicyclol attenuates tetracycline-induced fatty liver associated with inhibition of hepatic ER stress and apoptosis in mice. Can. J. Physiol. Pharmacol..

[bib0033] Zhang Y., Chang Y., Yang T., Wen M., Zhang Z., Liu G., Zhao H., Chen X., Tian G., Cai J., Wu B., Jia G. (2020). The hepatoprotective effects of zinc glycine on liver injury in meat duck through alleviating hepatic lipid deposition and inflammation. Biol. Trace Elem. Res..

